# Attitude of the Dentist Towards His Patient

**Published:** 1895-07

**Authors:** R. G. McLaughlin

**Affiliations:** Toronto.


ARTICLE VIII.

### ATTITUDE OF THE DENTIST TOWARDS HIS PATIENT.

BY R. G. MCLAUGHLIN, D. D. S., TOROTO.

Read before the Toronto Dental Society.

In this progressive age, when we are making such rapid strides in practical improvements, our professional energies are naturally occupied in keeping pace with the new ideas that are ever being advanced in the practical part of our work. But is it not wise to sometimes call a halt in this race with crowns, bridge-work, abscesses, and the numerous other cases ever before us, and just for a moment take a glance at ourselves personally, and ask how we are carrying ourselves before the world—how our professional standing compares with that of our brethren round about us? What is our general attitude and demeanor before our patients? and last, but not of least importance, with what amount of respect (professionally and otherwise) do our regular patients, month after month, deign to treat us.

These are questions that ever and again come to the thoughtful and ambitious man, whatever his profession may be. But you naturally ask, "What has all this to do with the success or failure of the modern and practical dentist?" I answer at once, a great deal. If there is a calling in life in this age in which good manners, gentlemanly address, quiet dignity and professional self-respect form many of the rungs in the ladder of success, that calling is surely dentistry. Here it is where the true gentleman stands head and shoulders above his confreres; here it is where the really educated man shows to best advantage, and here especially is where the man of refined tastes, and whose every word and action is filled with harmony, is sought after by the choicest and best in the land.

But if we are to come from the general to the particular, we must take ourselves to the operating room, and follow the dentist as he is receiving, day after day, his ever-changing classes of patients, for here, if at any place, the man's true professional spirit is brought to the surface. In his every dealing with his patient the dentist should never for a moment lose sight of his self-respect. There should ever be beneath the surface that quiet dignity of manner and unruffled temper which are ever indicative of the true gentleman. You may be assured your patients will not be slow in appreciating your worth in this particular and deporting themselves towards you accordingly.

Again, closely allied with what I have just said, and of no less importance, is the professional spirit generally displayed by the dentist in the discharge of his daily duties. Right here, I believe, is our great vantage ground in gaining the confidence and professional respect of our patients and right here is the point where the patient can and must be educated to a proper appreciation of the standing of the dentist among the professional men of the world. To realize how this educating process is being carried forward or retarded by the different members of our profession we have but to look to the general deportment of the various patients who for the first time come to us for treatment from the different parts of the city or Province. They have come from different offices, from different operators, from men ranging in professionalism from the lowest-bred quack to the ideal professional spirit. And how long does it take the patient to reveal to any of us by her every word and action the psofessional calibre of her last regular dentist?

You meet now the distrustful patient, who appears to have not the slightest confidence in your advice for the preservation of her teeth, and when told that certain molars and bicuspids need immediate attention, will not be convinced till mirrors are produced and the cavities seen with her own eyes—a sure indication that her last dentist had not succeeded in gaining her confidence. What is the remedy? Here, I think, it is: man before method. Differently constituted patients need different treatment; but, in general, I think it is better to quietly impress on the patient the fact that such is the condition then existing that such and such remedial operations are necessary, but she, as the patient; is at full liberty to decide whether the teeth are to be preserved or not.

Again, we all occasionally meet the *dictating* patient—the one who knows all about it, and who only looks on the dentist as a mere mechanical assistant, whose every operation must be under her direct supervision. How are we to treat such a patient? Certainly it is sometimes a difficult matter to solve. The indications strongly point to the patient being pampered and spoiled either by the special indulgence and want of self-confidence in her former dentist. Just how to give such a patient to understand that such a directory manner on her part is entirely out of place, is a problem that each practitioner must settle for himself. Certainly, if the dentist be of the right professional material and possessed of sufficient confidence and ability, most such refractory patients will not be long in being educated to the knowledge of their proper behavior in the dental chair.

We might go on multiplying these examples that daily come to us from a badly educated public; but enough has been said to show us where we stand and whither there is danger of us drifting. Yet each practitioner, wherever he may be placed, has the opportunity of training for himself, if he be possessed of the proper professional spirit, a class of patients who will daily manifest to him such general respect and professional confidence that will make his hourly tasks more genial and full of sunshine than they otherwise would be.

But there is another part of this subject that I am not willing to overlook. The dentist, in his usual round of probing, drilling and burnishing, should ever show towards his patient a spirit of practical sympathy. Once your victim fully understands that in every thrust and twinge of pain you are fully alive to the fact that you are operating on live tissue, you will have gone a long way in winning her confidence and making of her a lasting friend. How naturally we become more and more hardened as we grow older and busier in the work! How we soon come to drill and burr into a sensitive tooth with the same cold deliberation we might show in drilling into so much wood or stone! How apt we are, especially in our busier moments, to become irritable and impatient if the poor cringing and terrified creature, when taking the chair on the first occasion, is occupying too much of our precious time in getting over her paroxysm of fear and mustering sufficient courage to open wide her mouth. Let me tell you, the best practical lesson I, for one, get in exercising more patience in such cases is once in a while occupying the chair myself. No doubt, we are liable to become so absorbed in the success or failure of the operation in hand that we, for the time being, lose sight of the sufferings of the patient. In this respect I think mistakes are frequently made which prove far reaching in their effects; for example: a young girl of fifteen is in the dental chair for the first time, and the operation in hand is a gold-filling. The dentist has his whole mind occupied in completing a perfect operation, and after an hour's patient toil on his part, accompanied throughout by moanings, pleadings and hysterical sobs from the patient, the operation is completed and a perfect gold filling is the result. We now ask ourselves, Has this hour's work been a success? In one sense, Yes; in a greater sense, No. The operator has certainly succeeded in inserting a beautiful gold-filling, and so saving from the ravages of decay that one tooth; but, at the same time, he has also succeeded in so worrying and terrifying that delicate patient that she will never again willingly enter a dental office, and consequently the remaining twenty eight or thirty teeth may through the effects of that one operation be sacrificed. Now, the operator's first care in such a case should be to perform at that sitting as delicate and painless an operation as the present preservation of the tooth would permit. A temporary filling might answer the purpose fully as well for the time being, and in after months, when the patient had gained more courage and nerve-power, the gold work, if necessary, could be accomplished at a less sacrifice.

Let me say, in conclusion, that our whole aim in this matter should be to accomplish the highest class of work with the minimum amount of pain, and in this advanced age in dentistry our patients are beginning to expect from us, and to some extent properly so, that our operations should be, in general, if not absolutely painless, as nearly so as the circumstances will permit.—*Dominion Dental Journal.*

